# Predators and nutrient availability favor protozoa-resisting bacteria in aquatic systems

**DOI:** 10.1038/s41598-018-26422-4

**Published:** 2018-05-30

**Authors:** A. Andersson, J. Ahlinder, P. Mathisen, M. Hägglund, S. Bäckman, E. Nilsson, A. Sjödin, J. Thelaus

**Affiliations:** 1 Department of Ecology and Environmental Science, Umeå University, SE-901 87 Umeå, Sweden; 2 Umeå Marine Sciences Centre, SE-905 71 Hörnefors, Sweden; 3 Division of CBRN Defence and Security, Swedish Defence Research Agency, FOI, SE-901 82 Umeå, Sweden

## Abstract

The long co-existence of bacteria and protozoa has led to the development of bacterial protozoa resistance strategies, which are suggested to serve as drivers for the evolution of pathogenic bacteria. However, the ecological mechanisms underpinning selection for protozoa-resistance in aquatic bacteria are poorly known. To assess the role of nutrient availability and predation-pressure on selection for protozoa-resisting bacteria (PRB), an enrichment-dilution experiment was designed using laboratory microcosms containing natural lake water. PRB was monitored by screening 16S rRNA amplicon sequence data for reads assigned to bacteria that previously has been shown to resist degradation by amoebae. To estimate the effects of the microbial food web dynamics (microscopy of; heterotrophic bacteria, phytoplankton, protozoa and rotifers) and physicochemical variables on the PRB abundance in the study system, a joint species distribution modelling approach was used. The predation-pressure (ratio between predator and bacterial biomass) had a positive effect on the abundance of the PRB genus *Mycobacterium*, while perturbation (enrichment and dilution) favored the PRB genus *Pseudomonas* that dominated the bacterial community in the disturbed systems. Our results show that PRB with different ecological strategies can be expected in water of high and intermediate nutrient levels and after major disturbances of an aquatic system.

## Introduction

The potential for transmission of pathogenic bacteria from environmental reservoirs to susceptible hosts depends on the pathogens’ environmental distributions and the hosts’ behaviour. Knowledge of pathogens’ environmental persistence is limited, largely because it is difficult to study these microbes in their natural environments. However, a better understanding of pathogens’ long-term fates in natural ecosystems is needed to accurately assess risks of exposure and design effective strategies for responding to emerging diseases^1,2^.

The long co-existence of bacteria and bacterivorous protozoa in aquatic and terrestrial ecosystems has led to the evolution of antipredator strategies in many bacterial groups^3^. Bacteria that can survive protozoan predation, *i.e*. protozoa-resisting bacteria (PRB), frequently exhibit traits such as morphological adaptation (e.g. elongation, aggregation, and filament or biofilm formation), increased growth rates, motility, toxicity and ability to replicate or avoid degradation in eukaryotic cells using specific outer membrane structures^4–9^. Interactions between bacteria and bacterivores have been suggested to serve as a driver or selective force for pathogen evolution^3,10–14^. Pathogenic bacteria contain virulence factors that enable them to colonize a niche in their hosts, evade or suppress the host’s immune response, enter and exit host cells, or obtain nutrition from the host. Several of these abilities are similar to those of PRB; it is widely believed that many traits that render bacteria pathogenic in susceptible hosts were shaped by evolutionary forces outside the context of human-pathogen interactions, and should be seen as colonization factors that produce “accidental virulence”^15^.

Bacteria in natural aquatic systems are exposed to diverse stressors that vary over time and over spatial scales. In addition to protozoan predation and viral lysis, aquatic bacterial communities are exposed to bottom-up forces such as limited nutrient availability^16–18^. Consequently, these communities experience highly variable conditions, creating fluctuations in their selection dynamics *i.e*. variation in the bottom-up and top-down control of the microbial communities^19,20^. The heterogeneity of the aquatic environment may create niches that enable the co-existence of bacteria with different defense traits. Therefore, PRB could theoretically occur in any aquatic system. However, the ecological mechanisms underpinning selection for protozoa-resisting bacteria with varying ecological strategies are poorly known.

We performed a controlled experiment with the aim to elucidate effects of bottom up (nutrient level) and top-down forces (predation) on the occurrence of PRB genera in natural aquatic microbial communities. The presence of PRB was monitored by amplicon sequencing of the 16S rRNA gene. PRB were defined as amoeba-resisting bacteria^13^, which comprises bacteria shown to resist amoeba degradation with varying ecological strategies (amoeba associated and free-living). We hypothesized that increased nutrient load would increase the predation-pressure on the bacteria, leading to selection for PRB.

## Results

### Dynamics of nutrients, plankton and predation-pressure on bacteria

**Figure 1:**
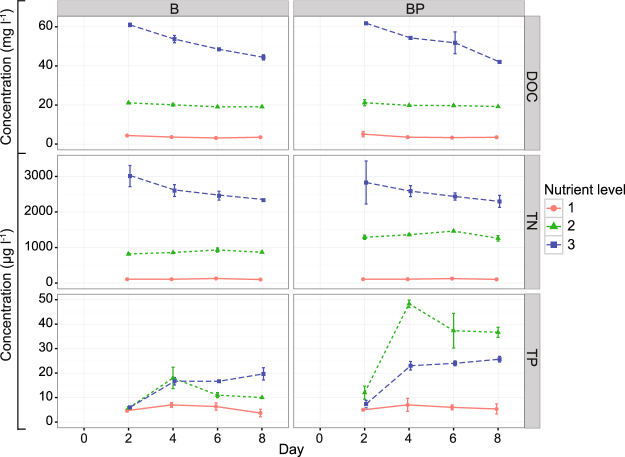
Concentrations of dissolved organic carbon (DOC), total nitrogen (TN) and total phosphorus (TP) during the experiment. Treatments: B = bacteria, BP = bacteria + predators and nutrient level = 1 (diluted), 2 (*in situ*), or 3 (enriched). Error bars indicate standard deviations.

The dissolved organic carbon (DOC) and total nitrogen (TN) concentrations for the highest and lowest nutrient levels (levels 3 and 1) differed ~20–30-fold, while TP concentrations differed by a factor of ~10 (Fig. 1). At nutrient level 3, the DOC and TN concentrations decreased over time, but at lower levels (2 and 1), DOC and TN were stable throughout the experiment. TP increased between days two and four and then remained relatively stable.

**Figure 2:**
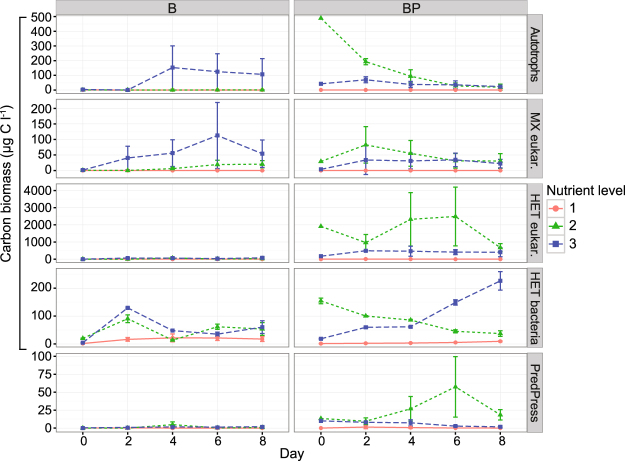
Carbon biomasses of autotrophic plankton (Autotrophs), mixotrophic plankton (MX eukar.), heterotrophic predators (HET eukar.), heterotrophic bacteria (HET bacteria) and predation pressure on bacteria (PredPress) during the experiment. Treatments: B = bacteria, BP = bacteria + predators and nutrient level = 1 (diluted), 2 (*in situ*), or 3 (enriched). Error bars indicate standard deviations.

**Figure 3:**
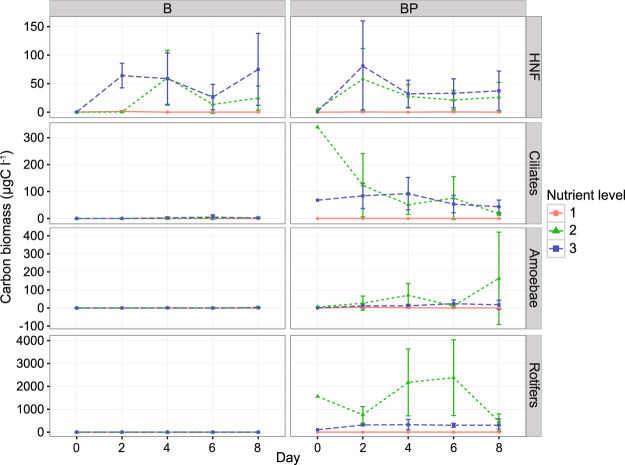
Biomass concentrations of heterotrophic predators, *i.e*. heterotrophic nanoflagellates (HNF), ciliates, amoebae and rotifers during the experiment. Treatments: B = bacteria, BP = bacteria + predators and nutrient level = 1 (diluted), 2 (*in situ*), or 3 (enriched). Error bars indicate standard deviations.

The biomass of autotrophic and mixotrophic plankton was lowest at nutrient level 1 and highest at levels 2 and 3 (Fig. 2). Initially their biomasses were highest in BP2, but decreased over time. Despite incubation in darkness, the biomasses of autotrophic and mixotrophic plankton increased in B3. Heterotrophic predator biomass were high in BP2, intermediate in BP3, and very low or absent in BP1 (Fig. 2). Heterotrophic predators in the BP2 and BP3 included rotifers, amoebae, ciliates and heterotrophic nanoflagellates (HNF) (Fig. 3). The B treatments were generally free of predators, although HNF were detected in B2 and B3 (Fig. 3).

The bacterial biomass increased steadily over time in BP3, but gradually decreased from an initially high level in BP2 (Fig. 2). Similar patterns occurred in B2 and B3. Conversely, the bacterial biomass remained low throughout in both nutrient level 1 treatments (B1 and BP1).

Initially the predation-pressure on bacteria in BP2 and BP3 were equally high (Fig. 2). However, over time the pressure increased in BP2. Towards the end of the experiment, the predation-pressure on bacteria was 20 times higher in BP2 than in the other treatments.

### Bacterial community composition

The 16s rRNA amplicon sequencing yielded 12,509,951 reads, 11,176,076 (89.3%) of which remained after OTU picking. The reads were clustered into 6,463 OTUs. After filtration, 10,882,635 reads remained, clustered into 544 OTUs; the number of reads per sample ranged from 19,471 to 243,297 (Appendix A). The rarefaction curves, representing the estimated diversity within the different treatments, reached an asymptote at a sequencing depth equal to the minimum number of reads per sample (Suppl. Figure 2). This indicates that the sequencing depth was sufficient for further analysis and most of the samples’ expected diversity was captured.

Eleven major phyla (including sub-classes of Proteobacteria) were identified: Actinobacteria, Alphaproteo-bacteria, Bacteroidetes, Betaproteobacteria, Cyanobacteria, Deltaproteobacteria, Firmicutes, Gammaproteobacteria, Gemmatimonadetes, Planctomycetes and Verrucomicrobia (Suppl. Figure 3). Sub-classes of the phylum Proteobacteria were included because most of the reads (39.0–97.8% per sample) were classified to that phylum. Betaproteobacteria was the dominant group at the lowest nutrient levels (1 and 2), but Gammaproteobacteria dominated at nutrient level 3.

The bacterial community’s alpha diversity was higher in the undisturbed *in situ* cultures (nutrient level 2) than in the diluted or enriched treatments (estimate 5.25, SE 0.72, p = 2.57E-9). Presence of heterotrophic predators resulted in higher alpha diversity in BP2 and BP3 than in other treatments (estimate 4.72, SE: 0.72, p = 9.74E-9).

### Factors promoting the occurrence of protozoa-resisting bacteria (PRB)

**Figure 4:**
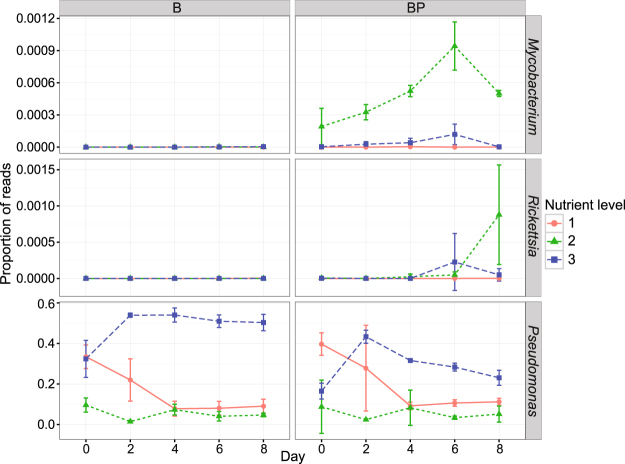
Proportion of reads assigned to protozoa-resisting bactreial (PRB) genera *Mycobacterium*, *Rickettsia* and *Pseudomonas* during the experiment. Treatments: B = bacteria, BP = bacteria + predators and nutrient level = 1 (diluted), 2 (*in situ*), or 3 (enriched). Error bars indicate standard deviations.

OTUs associated with three genera of PRB were identified from the 16S rRNA sequence data: *Pseudomonas* (Gammaproteobacterium), *Rickettsia* (Alphaproteobacterium) and *Mycobacterium* (Actinobacterium). The proportion of reads assigned to *Mycobacterium* and *Rickettsia* increased over time in BP2 (Fig. 4), which had the highest predation-pressure (Fig. 1). However, these PRB genera constituted a relatively small proportion of the total bacterial reads (~0.1%) in all samples. *Pseudomonas* was promoted in the enriched treatments B3 and BP3 (Fig. 4). In these samples, *Pseudomonas* quickly became dominant, constituting 30–50% of the total bacterial reads. A PCA biplot of measured variables indicated that *Pseudomonas* was linked to high DOC and TN concentrations and low predation-pressure (Suppl. Figure 4A), while *Mycobacterium* and *Rickettsia* were linked to heterotrophic nano- and microplankton and high predation-pressure, TP and alpha diversity. Pearson correlation analysis showed that *Pseudomonas* correlated negatively with *Rickettsia* and *Mycobacterium* (Suppl. Figure 4B).

**Figure 5:**
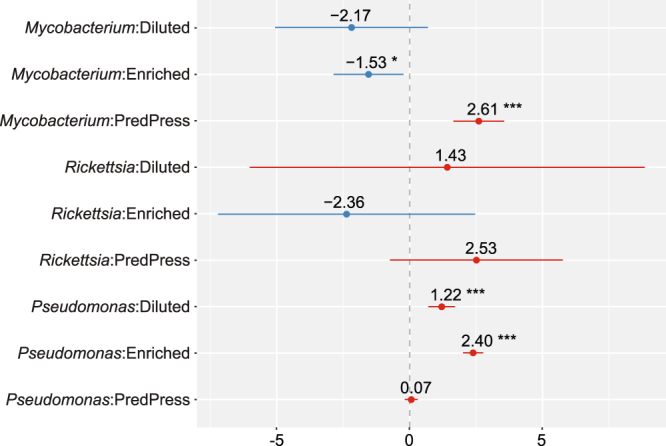
Summary statistics of the joint species distribution model (JSDM) analysis for experiment days 2–8, including effect sizes and 95% effect size confidence intervals for the nutrient and predation pressure (PredPress) parameters. Blue and red colors indicate negative and positive effect directions, respectively. Statistical significance at the α = 0.05, α = 0.01 and α = 0.001 levels is denoted by *, ** and ***, respectively. Treatments: Diluted (nutrient level 1), Enriched (nutrient level 3).

The JSDM analysis showed that predation-pressure had a strong positive effect on *Mycobacterium* abundance ($\hat{\beta }$_51_ = 2.61; 95% CI: 1.68, 3.55) (Fig. 5). This corresponds to a greater than 13-fold predicted increase in abundance if the predation-pressure ratio increased by 50% while all other conditions were unchanged. The same analysis revealed a negative association for *Mycobacterium* abundance with both dilution and enrichment compared to the undisturbed condition (Fig. 5). The association with dilution was weak ($\hat{\beta }$_211_ = −2.17; 95% CI: −5.05, 0.71) but that with enrichment was more obvious ($\hat{\beta }$_213_ = −1.53; 95% CI: −2.85, −0.22). The relatively rare occurrence of *Rickettsia* resulted in wide estimated 95% CI ranges for all parameters: none of the corresponding parameters was statistically significant. However, given that *Rickettsia* was only detected in predator-rich samples, predation-pressure seemed to be a favorable predictor ($\hat{\beta }$_52_ = 2.53; 95% CI: −0.71, 5.76). The biomasses of both amoebae (Fig. 3) and *Rickettsia* peaked on the last day of the experiment (Fig. 4), suggesting potential co-occurrence. Both dilution and enrichment had strong positive effects on the abundance of *Pseudomonas* ($\hat{\beta }$_231_ = 1.22; 95% CI: 0.73, 1.70 and $\hat{\beta }$_233_ = 2.40; 95% CI: 2.04, 2.76). This implies that going from the undisturbed *in situ* state to the enriched state without changing any other predictors would increase the *Pseudomonas* abundance ten-fold. Similarly, a change from the undisturbed *in situ* state to the diluted state would increase abundance three-fold. Predation-pressure was shown to be less important ($\hat{\beta }$_53_ = 0.07; 95% CI: −0.15, 0.30).

The JSDM analysis revealed a negative correlation between the occurrence of *Pseudomonas* and the two other PRB genera (r = −0.27 and r = −0.30 for *Mycobacterium* and *Rickettsia*, respectively), but there was a positive correlation between *Mycobacterium* and *Rickettsia* (r = 0.60). These inferred correlations were obtained after adjusting for treatment effects, time and replicate dependencies, and are therefore lower than the corresponding Pearson correlations (see Suppl. Figure 4B). There was, on average, a predicted decay in the abundance of PRB in the diluted samples over time that manifested as a negative slope effect. In all other cases, the predicted abundance of PRB genera increased over time. A JSDM analysis of only the last-day data resulted in parameter estimates that almost always included zero within the 95% CI (results not shown). This result suggested that all the data from day’s two to eight should be included in the modelling. Excluding rotifers in the calculation of predation pressure on bacteria, yielded similar results in the JSDM analysis (Suppl. Figure 5). However, one difference was that the predation pressure had less distinct influence on the occurrence of *Mycobacterium*, indicated by a wider 95% confidence interval of the regression coefficient (Suppl. Figure 5).

## Discussion

Contradicting our hypothesis, the predation-pressure did not increase in parallel with the nutrient load in natural lake water. This was unexpected because a previous field study identified a weak but general increase in predation-pressure with increasing productivity^21^. Here, the predation-pressure increased >1000-fold from the diluted to the undisturbed *in situ* state, and >100-fold from the diluted state to the highest nutrient load. The DOC concentrations in the experiment ranged from ~3 to 50 mgC l^−1^, with carbon concentrations in the diluted, *in situ*, and enriched treatments corresponding to those of drinking water or oceanic water, eutrophied waters and hypertrophic aquatic systems^22–24^, respectively. Predation-pressure had a strong positive effect on *Mycobacterium*, while nutrient availability had a negative effect on this bacterial genus. The opposite was observed for the opportunistic *Pseudomonas*, which was greatly promoted by nutrient enrichment. Our results thus imply there is a threshold beyond which the predation-pressure on bacteria does not increase further. This may be due to changes in the bacterial community composition that could arise if predation-resistant bacteria have negative effects on their predators.

*Mycobacterium* was favored by *in situ* conditions with nutrient concentrations matching those found under eutrophic conditions. The predator biomass, the predation-pressure on bacteria, and the alpha diversity of bacteria were all high under these circumstances. The genus *Mycobacterium* is divided into the tuberculosis and non-tuberculous complex, both of which have obligate or facultative intracellular lifestyles^25^. Species within the *Mycobacterium tuberculosis* complex (MTC) cause tuberculosis in mammals and humans, predominantly as a result of direct host-to-host contact^26,27^. Non-tuberculous mycobacteria (NTM) are responsible for community-acquired and focal health-care associated infections, and have been isolated from natural waters, water distribution systems, drinking water, soil, food, biofilms, aerosols and dust^28,29^. In keeping with previous studies, our results show that top-down control of the microbial community favors the occurrence of mycobacteria^25,30–33^. Rotifers and protozoa are both potential predators of bacteria. Omnivory is very common among both ciliates and rotifers^34–36^, so it is likely that they fed on both bacteria and HNF in the *in situ* and enriched treatments. In the present study, it is not clear whether the *Mycobacterium* were growing intracellularly in the protozoa or saprozoically (extracellularly), as shown for *Mycobacterium avium* in co-culture with amoeba^37^. Nevertheless, our results indicate that predatory protozoa have important effects on the persistence and dissemination of *Mycobacterium* in aquatic systems as a source of human infection^32^.

The obligate intracellular *Rickettsia* spp. appeared to be promoted by high predation-pressure, but this could not be proven by statistical analysis. *Rickettsia* spp. are obligate intracellular bacteria associated with eukaryotes, mainly arthropods, and range from being harmless to etiologic agent of severe disease in humans^38,39^. In our study, *Rickettsia* showed a marked covariation with *Mycobacterium*, indicating that they have similar ecological niches in the system. This suggests that both genera exist in association with protozoa and other predators, and are therefore favored under systems subject to strong top-down control. Notably, under *in situ* conditions, the abundance of both *Rickettsia* and amoebae peaked on the last day of the experiment (Figs 3 and 4). This may indicate that *Rickettsia* depend on amoebae as hosts^39,40^. It would be interesting to further elucidate if *Rickettsia* use specific species or groups of species as hosts in natural systems.

*Pseudomonas* spp. dominated the enriched treatment constituting 20–40% and 50–60% of the bacterial sequences in the presence and absence of protozoa, respectively. Bacteria from this genus can grow on a wide range of organic compounds due to their vast metabolic capabilities^41,42^. Consequently, they can colonize diverse environmental niches and species within this genus are among the most widespread opportunistic pathogens, causing infections in hosts including insects, plants, animals and humans^42^. Several *Pseudomonas* strains respond to grazing by inducing resistance traits such as colony and biofilm formation and activation of the type III secretion system, which mediates the secretion of effector toxins^43–46^. Pathogenic *Pseudomonas aeruginosa* has been shown to kill the amoeba *Acanthamoeba castellani* by producing toxins mediated by the type III secretory system^45,47^. The opportunistic ability to compete for nutrients and fast growth together with the ability to form biofilms and micro-colonies enables *Pseudomonas* to monopolize the bacterial community in disturbed systems. In our study, nutrient enrichment greatly increased the abundance of *Pseudomonas*, which in turn reduced the general predation-pressure on bacteria. This is consistent with previous findings showing that *Pseudomonas* is promoted in eutrophic and disturbed systems such as streams receiving effluents from hospital waste and fertilized soils^48,49^.

The definition of PRB is based on amoeba-resisting bacteria (ARB), i.e. bacterial taxa that resist uptake by amoebae and can survive, grow inside, and exit free living amoeba after internalization^13,38^. These ARBs include established pathogens such as *Legionella pneumophila*, *Mycobacterium avium*, *Francisella tularensis* and *Rickettsia* sp. Advancements in genome sequencing have made it possible to monitor the presence of traits associated with bacterial pathogenicity, i.e. virulence genes and pathogenicity islands, in complex environmental samples. Bacterial traits linked to pathogenicity (protein secretion systems, toxins and pathogenicity islands) are known to be widespread in marine bacteria, being present in up to 8% of oceanic metagenomic data^50^. Previous studies have related the presence of virulence genes to environmental factors such as productive waters, polycyclic aromatic hydrocarbons, and pH, but have not linked these genes to the occurrence of pathogenic bacterial taxa or associated them with infectious disease^50,51^. This suggests that bacterial gene homologues associated with virulence in humans commonly enhance persistence in natural environments. We therefore suggest that analysis of PRB genera, could be useful to gain knowledge on ecological drivers that influence the long-term fates of pathogens in natural ecosystems.

This study shows that the generative model may be useful in risk assessment frameworks because it integrates environmental data (i.e. microbial food web dynamics and physiochemical data) with predictive modelling of bacterial abundance in aquatic systems. We have shown that the relationship between organic load and predation-pressure on bacteria is non-linear: there seems to be a tipping point beyond which PRB with extracellular lifestyles start to control the predators. Above this threshold, disturbances enable opportunistic bacteria such as strains within the *Pseudomonas* genus to monopolize the system, causing very little energy to be transferred up the food web. Conversely, the high predation-pressure on bacteria associated with undisturbed *in situ* conditions promotes other types of PRBs’, including the slow-growing intracellular *Mycobacterium* spp. and *Rickettsia* spp., which depend on eukaryotic cells for survival and replication. Our results thus indicate that waters of all nutrient states can harbor PRB, but that bacteria with different ecological strategies can be expected in water of high and intermediate nutrient levels and after major disturbances of the aquatic system.

## Material and Methods

### Microcosm experiment

A microcosm experiment was performed using water from a small eutrophic lake in southern Sweden (Suppl. Material 1). The experiment consisted of six triplicated treatments, including three different nutrient levels (1 - diluted, 2- *in situ* and 3- enriched) with or without predators (BP and B, respectively) (Suppl. Figure 1). The dilution treatment was designed to mimic oligotrophic conditions, while the enrichment treatment was intended to simulate hypertrophic environments. Bacterial composition and plankton biomass were analyzed on day 0, 2, 4, 6 and 8, while chemical analyses were performed on day 2, 4, 6 and 8.

### Chemical analyses

TP and TN were measured using a Braan & Luebbe TRAACS 800 autoanalyzer, according to standard analytical methods^52^. Dissolved organic carbon (DOC) was analyzed in water filtered through a 0.22 µm Supor Membrane Syringe Filter (non-pyrogenic; Acrodisc®) and acidified to 18 mM HCl, final concentration. Samples were analyzed with a Shimadzu TOC-5000 instrument.

### Bacterial abundance and biomass

Samples for bacterial counts were preserved in 0.1% glutaraldehyde (final concentration), frozen at −80 °C^53^ and analyzed with a BD FACSVerse^TM^ flow cytometer (BD Biosciences). Samples were stained with SYBR Green I (Invitrogen) at a final concentration of 1:10 000^53^. As internal standard, 1 μm microspheres (Fluoresbrite plain YG, Polysciences) were added to each sample. The bacterial carbon biomass was calculated using a conversion factor of 20 fg C cell^−1^ according to Lee and Fuhrman^54^.

### Nano and microplankton abundance and biomass

Samples were preserved with alkaline Lugol’s solution (2% final concentration) and analyzed using Utermöhl technique. Briefly, samples (10 ml) were added to sedimentation chambers and plankton allowed to settle for 12 hours before being counted with a Nikon inverted microscope (TMS) using phase contrast. Nanoplankton were counted in one transect at 400 times magnification, while microplankton were generally counted in half a chamber at 100 times magnification. Generally >100 cells per sample were counted. Phytoplankton, protozoan, and rotifer biovolumes were calculated from cell geometries as described by Olenina *et al*.^55^ and Ruttner-Kolisko^56^. Carbon biomasses were calculated according to Menden Deuer and Lessard^57^. Plankton were grouped according to nutritional strategy: autotrophy, mixotrophy or heterotrophy.

Results of chemical analyses and carbon biomasses of heterotrophic bacteria, phytoplankton, and predators were visualized using the R package ggplot2, version 2.1.0^58,59^.

### DNA extraction

Four ml of each water sample were centrifuged at 16 000 × g for 1 h, 3.9 ml of the resulting supernatant were discarded, and DNA was extracted from the remaining volume using the SoilMaster DNA Extraction Kit according to the manufacturer’s recommendations for environmental water samples (Epicentre Biotechnologies, Madison, WI, USA). The resulting DNA pellet was resuspended in 60 µl of TE buffer and either frozen and stored or immediately subjected to PCR analysis. Sample preparation, PCR reaction preparation, and thermal cycling were performed in different rooms.

### Amplicon preparation and sequencing

Amplicons were generated targeting the V4 region of the bacterial 16S rRNA gene^60^. Briefly, the V4 region was amplified using primers F515 (5′-GTGCCAGCMGCCGCGGTAA-3′) and R806 (5′-GGACTACHVGGGTWTCTAAT-3′). Forward and reverse primers were modified to incorporate a 12 bp Golay error-correcting barcode that enabled sample multiplexing with both primers^60^. Amplicons were prepared in triplicate and the resulting PCR products were pooled and analyzed on 1% agarose gel pre-stained with GelRed (1:10,000, GelRed™ Nucleic Acid Gel Stain, Biotium). Concentrations were determined with a Qubit dsDNA BR Assay Kit on a Qubit fluorometer (Invitrogen). The amplicons were pooled at equimolar concentrations and purified using the UltraClean PCR Clean-Up Kit (MoBio), then sequenced on the MiSeq platform (Illumina) with 500 bp paired-end reagent kits according to the manufacturer’s recommendations (MiSeq System User Guide, Part # 15027617 Rev. C).

### Amplicon sequence analysis

The Quantitative Insights Into Microbial Ecology (QIIME) pipeline^61^, version 1.7, was used to process the sequenced data. Reads were quality-filtered based on their Phred scores and remaining adapter sequences were removed with Cutadapt^62^. Overlapping read pairs were merged using FLASH^63^. Merged reads were matched to the reference database Greengenes version 13.8^64^ and clustered into operational taxonomic units (OTUs) through a closed-reference OTU picking approach using UCLUST^65^. Reference sequences were obtained by searching Greengenes for OTUs with at least 97% similarity to the target sequences^66^. Taxonomies were assigned to all OTUs within the OTU-picking workflow, which also includes removal of chimeric sequences using uchime^67^. Source code of scripts used in this work can be found in Appendix B.

A representative set of sequences was obtained in which each OTU was represented by its reference sequence from Greengenes. The representative set was aligned using the PyNAST method^68^ to the Greengenes core reference alignment. A phylogenetic tree was inferred using an approximately-maximum-likelihood method implemented in the software FastTree 2.1.3^69^. Singletons and low abundant OTUs with frequencies below 0.005% of the total reads were removed^70^. OTUs observed in at least three samples were retained. Alpha diversity was determined using the metric of phylogenetic diversity^71^. Differences in alpha diversity between nutrient and protozoa levels were estimated using a linear model fitted within R.

PRB were identified as previously suggested by Bertelli and Greub (Amoeba-resisting bacteria, **13**). In short, the Bertelli and Greub definition includes; bacteria that has been isolated from amoebae or shown growing within amoebae, bacteria shown to resist amoebae phagocytosis *in vitro* and intracellular and fastidious bacteria^13^. Reads assigned to OTUs of PRB genera were extracted from the sequence data and concatenated at genus level for further analysis after removing singletons and low abundant OTUs.

### Statistical analyses

#### Predation-pressure

We tested whether the predation-pressure on bacteria was a key factor for the occurrence of PRB. Predation-pressure on bacteria was calculated in two different ways:

1. Ratio between the carbon biomasses of heterotrophic protozoa+eukaryotes and heterotrophic bacteria:
  $${{\rm{Bact}}}_{{\rm{PredPres}}s}=({{\rm{Protozoa}}}_{{\rm{Het}}}+{{\rm{Metazoa}}}_{{\rm{Het}}})/{{\rm{Bacterial}}}_{{\rm{Het}}}$$
2. Ratio between the carbon biomasses of heterotrophic protozoa and heterotrophic bacteria^72,73^:

$${{\rm{Bact}}}_{{\rm{PredPress}}}={{\rm{Protozoa}}}_{{\rm{Het}}}/{{\rm{Bacterial}}}_{{\rm{Het}}}$$

#### Principal component analysis

To visualize the treatments’ effects, principal component analysis (PCA) was performed using data from the last day of the experiment (day 8), and the R package ade4^74^. Variables included were proportion of reads assigned to PRB, TN, TP, DOC, alpha diversity, and carbon biomasses of heterotrophic bacteria, autotrophs, mixotrophs, heterotrophic protozoa, and metazoa. The alpha diversity and proportion of reads assigned to PRB were square root transformed, while the other variables were log transformed. If the data contained zeros, a value of 0.001 was added prior to log transformation. Furthermore, Pearson correlations between the proportions of reads assigned to different PRB on the last day of the experiment were visualized with a heat map.

#### Joint species distribution model

Inferential insights into effects of nutrient availability and protozoan predation on the abundance of PRB were drawn by fitting a joint species distribution model (JSDM)^75^: based on a generalized linear mixed model (GLMM) framework, implemented in the lme4 package (version 1.1-12) in R^76^. Total abundance of PRB sequence data was included as the (multivariate) response variable, while total sequence abundance was included as a covariate to control for differences in sampling depth. Thus, we modelled the 16S rRNA sequence data as compositional rather than absolute abundance data. To access residual correlations among PRB reads, a multivariate random intercept at each sample was included in the model. Nutrient level (diluted, *in situ* and enriched) was included in the model as a group-level predictor (coded with n = 1, 2, 3, respectively).

The model was set up hierarchically to adjust for dependencies over time and among replicates. Each treatment group (i.e. combination of experimental predictors, such as enrichment, dilution, and/or protozoan filtering) was treated as a random effect with varying intercept and slope: this constituted the second level of the hierarchy, which ensured that the time dependency was acknowledged. The replicates for a given combination of time point and treatment were modelled with a random intercept, and constituted the first level in the model. For each sample i = 1, …, 72, for each PRB j = 1, 2, 3, for each treatment group k = 1, …, 6, and for each combination of treatment and time point l = 1, …, 24, the multilevel model can be written as:

$${\rm{g}}({{\rm{m}}}_{{\rm{ijkl}}})={{\rm{\beta }}}_{{\rm{0i}}}+{{\rm{\beta }}}_{{\rm{1j}}}+{{\rm{\beta }}}_{{\rm{2jn}}}+{{\rm{\beta }}}_{\text{3k}[i]}+{{\rm{\beta }}}_{\text{4l}[i]}+{{\rm{x}}}_{{\rm{5i}}}\,{{\rm{\beta }}}_{{\rm{5j}}}+{{\rm{x}}}_{{\rm{6i}}}{{\rm{\beta }}}_{\text{6k}[i]}+{{\rm{u}}}_{{\rm{ij}}}$$

g() is the log link function defining the mean of the linear function of predictors, m_ijkl_ is the PRB abundance, β_0i_ is the effect of the total sample sequence abundance for sample i, β_1j_ is the intercept for the j:th PRB, β_2jn_ is the n:th nutrient level effect on PRB j, β_3k[i]_ is the varying intercept of the k:th treatment for sample i in treatment k, β_4l[i]_ is the varying intercept of the triplicate for group l, β_5j_ is the effect of predation-pressure on PRB j, β_6k[i]_ is the varying slope of treatment k at time t = 2, 4, 6, 8 days, x_5i_ is the estimated predation-pressure for sample i, and x_6i_ is the time point when sample i was collected. Varying intercepts and slopes (i.e., β_3k_, β_4l_, β_6k_) are assumed to be normally distributed with mean zero and variance among the PRB abundance in each treatment group, respectively. The residual u_ij_ is assumed to be multinormally distributed with zero mean vector and an unstructured covariance matrix. The predation-pressure predictor, x_5i_, was log-transformed and scaled to unit variance prior to the analysis. Default values of the parameters controlling convergence of the glmer function in the lme4 package was used. Confidence intervals (CI) were calculated using the Wald method. To visualize the estimated effect size of the regression coefficients, the R package sjPlot^77^ was used. The data for days two to eight of the experiment was used to fit the JSDM model.

### Data availability

Sequence data, data set and source codes in appendix A and B.

## Electronic supplementary material

Supplementary information
