# Supplementary material for: Predators and nutrient availability favor protozoa-resisting bacteria in aquatic systems
Source: Sci Rep. 2018 May 30;8:8415. doi: 10.1038/s41598-018-26422-4 (PMC5976761; doi:10.1038/s41598-018-26422-4)
Supplement: Supplementary file 1 — Supplementary information [file 41598_2018_26422_MOESM1_ESM.pdf]

Supplementary Information:

Title: Predators and nutrient availability favor protozoa-resisting bacteria in aquatic systems

By: Andersson A, Ahlinder J, Mathisen P, Hägglund M, Bäckman S, Nilsson E, Sjödin A, Thelaus J

Sampling of water: In August 2013, 60 liters of water were sampled from a small eutrophic lake in southern Sweden (Lat. 15.146716, Long. 59.268392). The water was collected at 0.5 m depth using a Ruttner sampler. The *in situ* temperature was ~20 °C, the dissolved organic carbon (DOC) concentration was 29 mg l^{-1}, total nitrogen (TN) was 1479 µg l^{-1}, total dissolved organic nitrogen (TDN) was 1177 µg l^{-1}, total phosphorus (TP) was 88 µg l^{-1} and total dissolved phosphorus (TDP) was 35 µg l^{-1}. The water was transported to the laboratory and the microcosm experiment began within 48 hours.

Supplementary Figure 1: Experimental design.

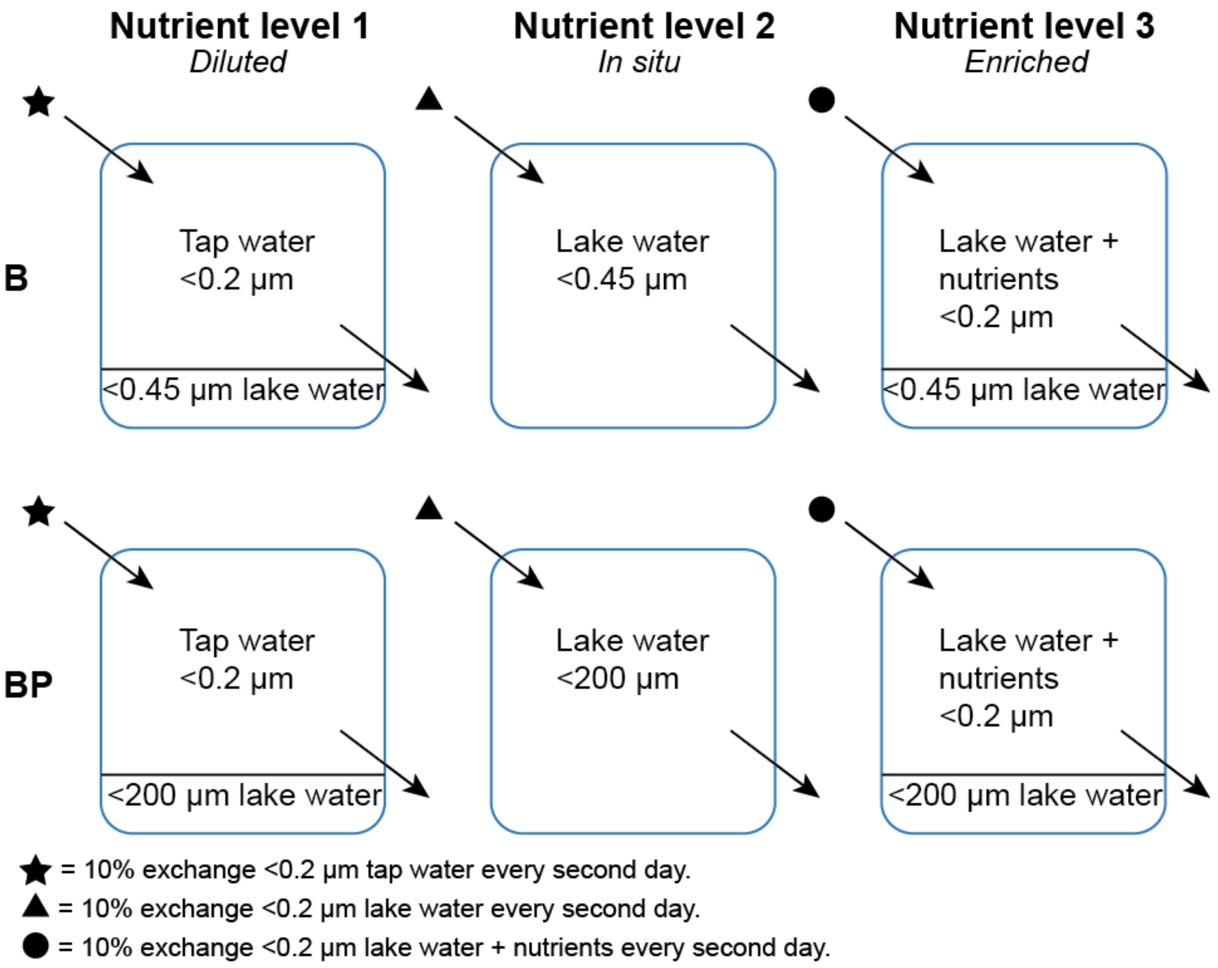

Treatments: B1 (bacterial nutrient level 1, diluted) - lake bacteria (obtained by 0.45 µm-filtering lake water) were incubated in low nutrient water (0.2 µm-filtered tap water). BP1 (bacteria + predators, nutrient level 1) – lake bacteria, protozoa, phytoplankton and rotifers (200 µm-filtered lake water) were incubated in low nutrient water (0.2 µm-filtered tap water). B2 (bacterial nutrient level 2, *in situ*) – lake bacteria (0.45 µm-filtered lake water). BP2 (bacteria + predators, nutrient level 2, *in situ*) – lake bacteria, protozoa, phytoplankton and rotifers (200 µm-filtered lake water. B3 (bacterial nutrient level 3, enriched) – lake bacteria (0.45 µm-filtered lake water) incubated in nutrient-enriched 0.2 µm-filtered lake water. BP3 (bacteria + predators, nutrient level 3) - lake bacteria, protozoa, phytoplankton and rotifers (200 µm-filtered lake water) incubated in 0.2 µm-filtered nutrient-enriched lake water.

Accomplishment: For the B1, BP1, B3, and BP3 treatments, 10% (by volume) of the appropriate inoculate (0.45 µm-filtered or 200 µm-filtered lake water) was mixed with 90 % (by volume) of the appropriate medium (0.2 µm-filtered tap water or 0.2 µm-filtered lake water). For the B2 and BP2 treatments, the "inoculates" constituted 100% of the water volume. Samples for the B3 and BP3 treatments were then enriched by adding organic carbon and inorganic nitrogen and phosphorus. The organic carbon solution consisted of glucose, galactose and sodium acetate (total addition 50 mg C l^{-1}), the inorganic nitrogen solution contained $NaNO_3$, (total addition 2.5 mg N l^{-1}), and the inorganic phosphorus solution contained NaH_2PO_4 (total addition 19 µg P l^{-1}). The CN additions were performed to mimic hypertrophic conditions (Zielinski et al. 2015). At the start of the experiment (day 0), 75% of the CN nutrients were added. After this initial boost addition, 8.3% of the nutrients were added on days 2, 4 and 6. P additions started on day 4 and were performed in a similar way as for C and N, but the additions were relatively small, resulting in values comparable to those in less productive freshwater systems (e.g. Andersson et al. 2013). The total volume of each microcosm was 1.3 liter and every second day 10% of the water was exchanged with new medium to stabilize the productivity in the different treatments. The flasks were incubated in darkness at room temperature (20 °C). Samples for analysis of bacterial composition, plankton biomass, and chemical factors were collected on days 0, 2, 4, 6 and 8.

References:

Andersson A, Jurgensone I, Rowe OF, Simonelli P, Bignert A, Lundberg E, Karlsson J. (2013). Can humic water discharge counteract eutrophication in coastal waters? *Plos One* 8 (4) doi:10.1371/journal.pone.00061293

Zielinski P, Grabowska M, Jekatierynczuk-Rudczyk E. (2016). Influence of changeable hydro-meteorological conditions on dissolved organic carbon and bacterioplankton abundance n a hypertrophic reservoir and downstream river. Ecohydrology 9: 382-395.

58 **Supplementary Figure 2.** Rarefaction curves of the DNA sequences.

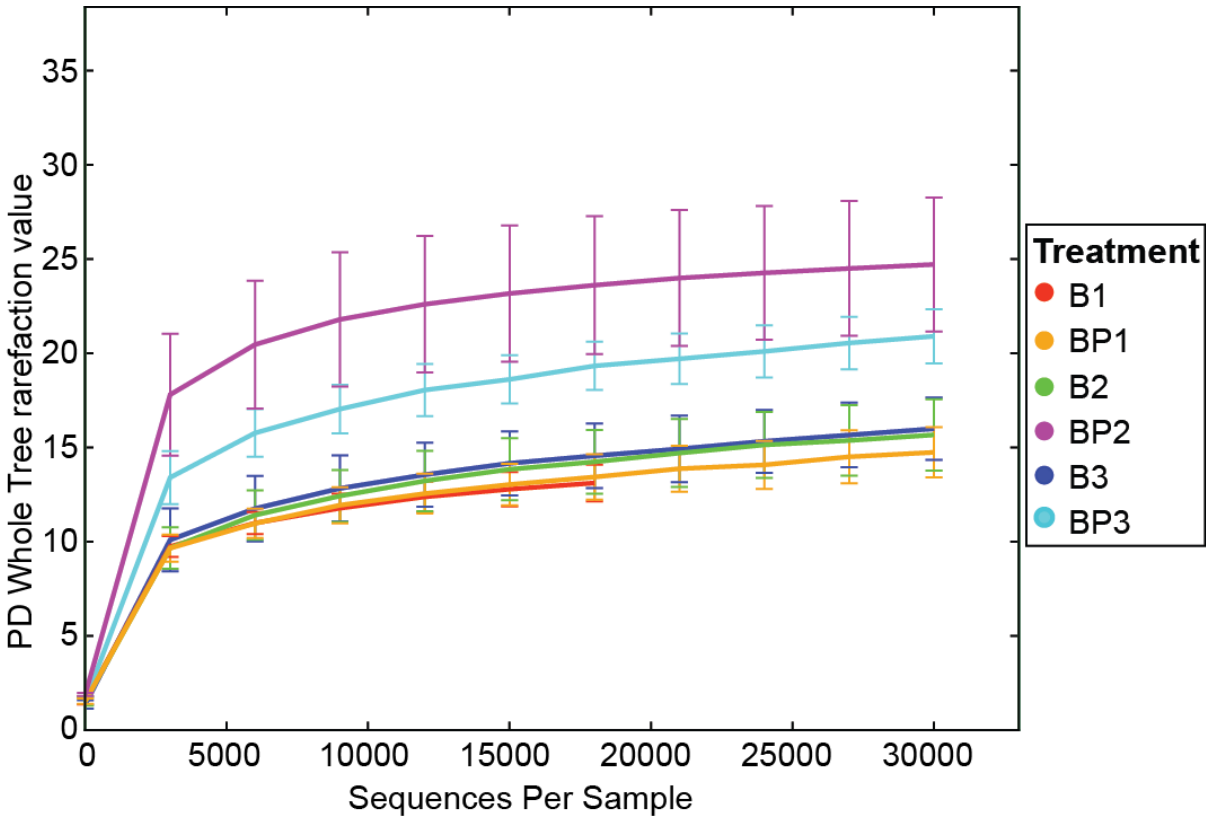

59

60

Supplementary Figure 3. Bacterial community composition at phylum level including sub-classes of Proteobacteria for each sample in the experiment. Treatments: B = bacteria, BP = bacteria+predators and nutrient level = 1 (diluted), 2 (*in situ*), or 3 (enriched).

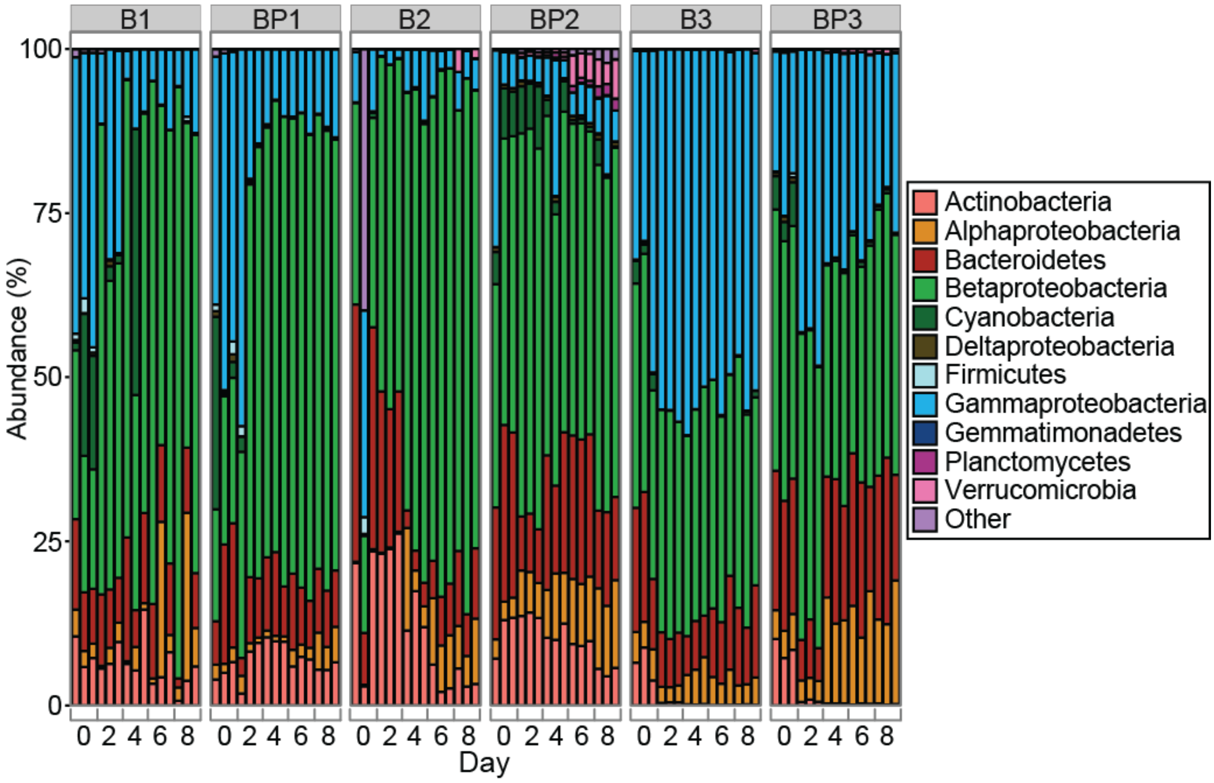

Supplementary Figure 4. A: Principal component analysis of the occurrence (proportions) of the PRB genera *Pseudomonas*, *Mycobacterium* and *Rickettsia* in relation to different environmental variables at the last day of the experiment*. B: Heatmap of the Pearson correlations between different PRB´s at the last day of the experiment. Abbreviations: Bacteria (heterotrophic bacteria), TN (total nitrogen), DOC (dissolved organic carbon), Autotrophs (prokaryotic and eukaryotic photosynthetic organisms), Mixotrophs (mixotrophic flagellates), Heterotrophs (heterotrophic predators), PredPress (ratio Heterotrophic predators:Bacteria) and TP (total phosphorus).

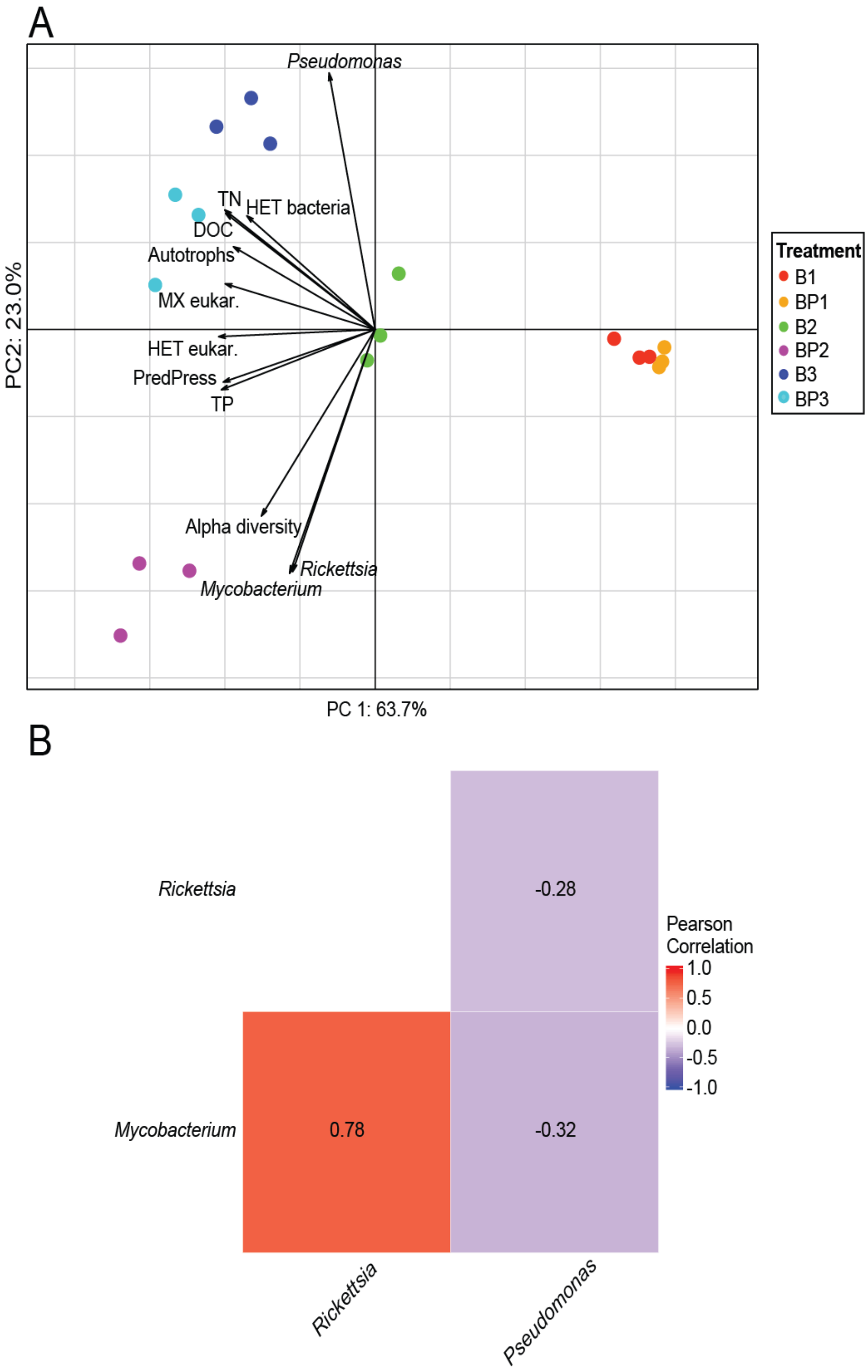

Supplementary Figure 5: Summary statistics of the joint species distribution model (JSDM) analysis for experiment days 2-8, including effect sizes and 95% effect size confidence intervals for the nutrient and predation pressure (PredPress, including only biomass of protozoa in the enumerator) parameters. Blue and red colors indicate negative and positive effect directions, respectively. Statistical significance at the α = 0.05, α = 0.01 and α = 0.001 levels is denoted by *, ** and ***, respectively. Treatments: Diluted (nutrient level 1), Enriched (nutrient level 3).

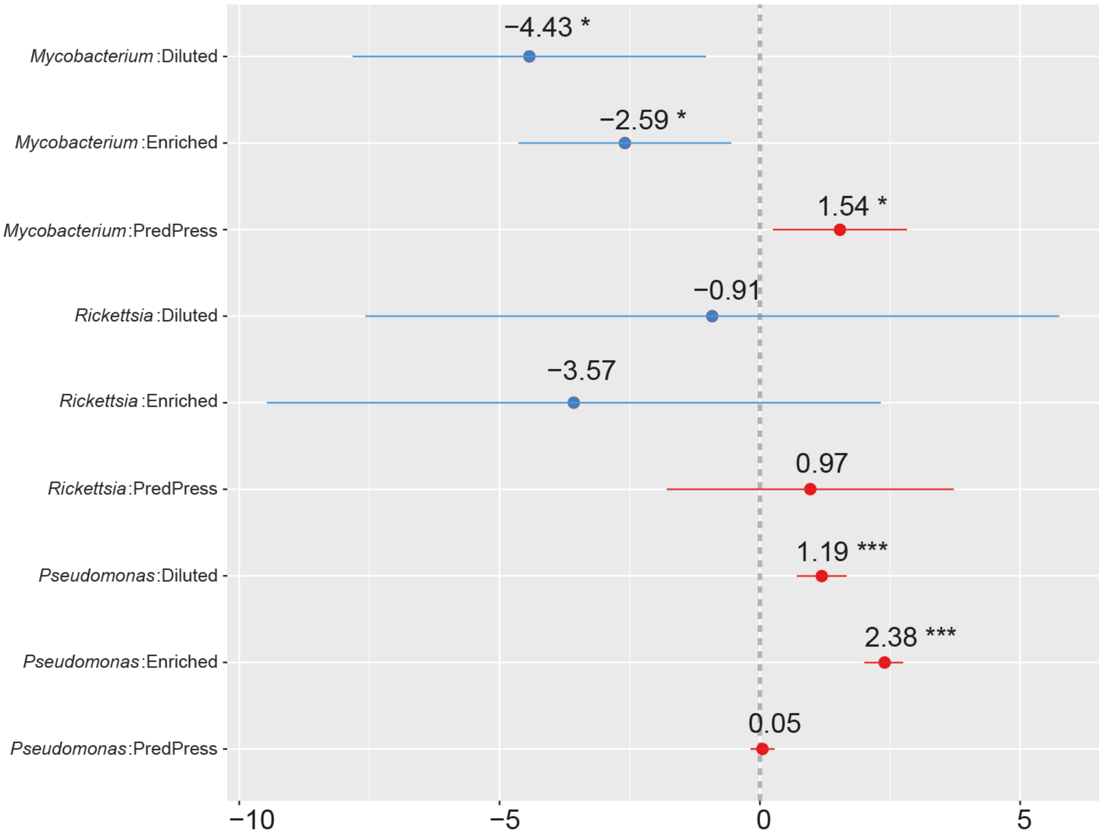

Appendix A. Sequence data can be accessed at the Short Read Archive ([http://www.ncbi.nlm.gov/sra)](http://www.ncbi.nlm.gov/sra), BioProject ID: PRJNA472564.

Appendix B. Data set and source codes can be accessed at https://github.com/FOI-Bioinformatics/Aquatic-ecosystems-at-risk-of-occurrence-of-pathogenic-bacteria
